# Protocol for Comparing Benzodiazepine-Ketamine and Benzodiazepine-Fentanyl Sedation in Phacoemulsification (BEKEF): A Double-Blind Crossover Non-Inferiority Clinical Trial Study

**DOI:** 10.7759/cureus.73328

**Published:** 2024-11-09

**Authors:** Rodrigo A Torres, Adriano C Faneli, Ricardo D. C Oliveira, Pablo Amado, Eduardo F Marback, Juliana F Marback, Larrie R Laporte, Cristina Muccioli

**Affiliations:** 1 Ophthalmology, HOBrasil Salvador, Salvador, BRA; 2 Medicine, Bahiana School of Medicine and Public Health, Salvador, BRA; 3 Ophthalmology, Federal University of Bahia, Salvador, BRA; 4 Anesthesiology, HOBrasil Salvador, Salvador, BRA; 5 Ophthalmology, Federal University of Sao Paulo, São Paulo, BRA

**Keywords:** cataract surgery, fentanyl, ketamine, phacoemulsification, sedation

## Abstract

Background: Cataract surgery, particularly phacoemulsification, often requires sedation alongside topical anesthesia to manage patient anxiety and discomfort. This protocol proposes a study comparing the efficacy of ketamine and fentanyl, both combined with benzodiazepine, as sedation options for patients undergoing bilateral phacoemulsification.

Methods: In a randomized, double-blind, crossover clinical trial, at least 48 patients scheduled for bilateral cataract surgery will receive fentanyl with midazolam for one eye and ketamine with midazolam for the other. Sedation levels will be assessed using the Ramsay Sedation Scale, and the SURG-TLX Scale will measure intraoperative difficulties. Secondary outcomes include anesthesia-related complications, pain scores, and patient satisfaction.

Results: The primary outcomes, utilizing the Ramsay Sedation Scale, will determine if ketamine combined with a benzodiazepine is non-inferior to fentanyl combined with a benzodiazepine for sedation during phacoemulsification. Secondary outcomes will provide insight into complications, patient and surgeon comfort, and overall satisfaction.

Conclusion: This trial will evaluate whether ketamine offers a safe and effective alternative to fentanyl for sedation in cataract surgery, potentially optimizing sedation protocols to enhance patient and surgeon experiences.

## Introduction

Cataract can be defined as the opacification process of the crystalline lens [1]. In 2010, cataract was the second leading cause of global moderate to severe visual impairment, accounting for approximately 33% of blindness cases [2]. When its presence begins to impact the patient's quality of life, affecting their daily activities, cataract surgery (phacoemulsification) is indicated as the sole efficient treatment for the disease [1,3].

For phacoemulsification, peri- or retrobulbar block anesthesia techniques can be applied. However, these are more associated with complications such as ocular globe perforation, optic nerve damage, retrobulbar bleeding, and injury to extrinsic ocular muscles [4]. Topical anesthesia, conversely, is less associated with complications arising from the injection technique required by blocks and is currently the preferred choice for ophthalmologists performing phacoemulsification. It is used in over 90% of uncomplicated cases, as reported in approximately 81% of 240 evaluated centers worldwide in 2021 by Rossi et al. [5,6]. General anesthesia, in turn, is only employed in specific cases, such as pediatric surgeries [4].

Despite its lower risk of complications, topical anesthesia does not provide complete analgesia to the iris, zonule, and ciliary body due to the blood-aqueous barrier, which limits the penetration depth of topical anesthetics, resulting in the possibility of pain during and after surgery [5,7,8]. Additionally, fear and anxiety are commonly reported emotions among cataract surgery patients, primarily driven by the fear of vision loss or deterioration and experiencing pain during the procedure [9]. It is also common for patients undergoing topical anesthesia to have visual experiences during surgery (flashes, appearance of shadows, etc.), which can further trigger fear and anxiety [9]. To address these factors, an option is to administer mild sedation concurrently with topical anesthesia, as Erdurmus et al. noted, enhancing patient and surgeon satisfaction with the surgery [1,4,9,10].

The primary objective of sedation is to minimize patient anxiety without increasing the surgical risk, maintaining the highest possible level of safety [11]. The goal is to alleviate fear, anxiety, and procedure-related pain while preserving the patient's ability to communicate with the surgeon during the surgery [11]. When sedation is employed, the ideal approach for most ophthalmic surgeries, including phacoemulsification, is to maintain the patient in a state of minimal sedation. As defined by the American Society of Anesthesiologists (ASA), this state is induced by medication and characterized by cognitive function and coordination possibly being affected, while cardiovascular, ventilatory function, and the ability to respond to verbal commands remain unchanged [11,12].

To maintain patient safety during sedation, it is crucial to keep their respiratory function stable [12]. If respiratory function and consciousness levels are preserved, cardiovascular depression secondary to sedation becomes highly unlikely [12]. Therefore, supplemental oxygen should be provided to every patient undergoing intravenous sedation, and the minimal required monitoring consists of an electrocardiogram (ECG), pulse oximeter, and a non-invasive method to monitor blood pressure [12]. Clinical observation of the patient during the procedure can also aid in monitoring, although it is more subjective and less validated and reliable [11]. A tool that can be utilized for this purpose is the Ramsey Sedation Scale, which classifies the patient into six sedation levels based on their general state and ability to respond to commands [11].

Fentanyl is a lipophilic opioid, with its maximum effect occurring 10-20 minutes after intravenous (IV) injection, lasting approximately 30 minutes [13]. At analgesic doses (2 to 10 micrograms per kilogram) and anesthetic doses (30 to 100 micrograms per kilogram), it rarely induces significant cardiovascular depression [13]. However, being a cumulative drug and stimulating the area postrema of the medulla, it may lead to nausea and vomiting [13]. Excessive repeated administration or use of high doses can result in prolonged respiratory depression [13]. In a randomized clinical trial in 2002, Aydin et al. observed increased patient and surgeon satisfaction with the use of IV fentanyl combined with topical anesthesia in cataract surgeries [14]. Again, in 2023, an observational study concluded that the use of fentanyl (this time in combination with propofol) was effective in achieving appropriate sedation levels safely and reaching acceptable patient satisfaction levels [15]. In 2012, Sanri et al. found that the drug, when combined with etomidate, compared favorably with ketamine combined with etomidate, showing fewer respiratory adverse effects and a more suitable hemodynamic profile [16]. However, it's essential to note that this prospective observational study focused on emergency room procedures, not specifically on ophthalmic surgeries [16].

Ketamine is another lipophilic drug with potent analgesic effects, effective even at low doses (10-20 mg) [11,13]. It is a sympathomimetic capable of maintaining stable blood pressure and respiration but is associated with tachycardia, hypertension, and increased intraocular pressure at high doses [11]. It can induce "dissociative anesthesia," characterized by profound analgesia, disconnection from the physical environment, and superficial sedation [13]. It can also produce negative dreams and hallucinations, an effect that diminishes when combined with a benzodiazepine [11]. Additionally, nausea and vomiting are possible adverse effects [11]. A study by Cugini et al. concluded that the use of intravenous low-dose ketamine (0.3 mg/kg) combined with diazepam (benzodiazepine) during phacoemulsification with block anesthesia had a satisfactory analgesic effect. It did not increase intraocular pressure, maintained systemic blood pressure and ventilation at adequate levels, and was not associated with nausea or hallucinations in patients [17]. A clinical trial published in 2015 demonstrated that, during cataract surgeries with topical anesthesia combined with sedation, ketamine combined with fentanyl achieves similar levels of patient satisfaction and hemodynamic stability as propranolol without additional medications. However, it may cause more nausea and vomiting [18].

Fentanyl is a widely used drug, whether combined or not with other medications, to assist in sedating patients undergoing phacoemulsification under topical anesthesia. Ketamine also appears to be an effective and safe option, especially when combined with another medication. Currently, there seems to be a lack of studies in the literature comparing the use of fentanyl combined with a benzodiazepine to ketamine combined with a benzodiazepine as an adjuvant sedative during phacoemulsification under topical anesthesia.

In this context, given the lack of studies on ketamine use in cataract surgery and existing evidence indicating fewer respiratory adverse effects and a more favorable hemodynamic profile compared to fentanyl [16], this study aims to determine whether ketamine combined with a benzodiazepine provides non-inferior sedation to fentanyl combined with a benzodiazepine during phacoemulsification. Through the evaluation of sedative effectiveness, safety profile, and patient and surgeon satisfaction, the study seeks to offer evidence that ketamine, when combined with a benzodiazepine, could serve as an equally effective alternative to fentanyl, with potential advantages in managing pain and anxiety during cataract surgery.

## Materials and methods

Design

This study is a prospective, randomized, double-blind, crossover, non-inferiority clinical trial designed to compare the sedative effects of two drug regimens during phacoemulsification surgeries. The regimens under comparison are benzodiazepine combined with fentanyl versus benzodiazepine combined with ketamine. Each patient will serve as their own control, receiving one sedative combination for the first eye surgery and the alternate combination for the second eye. The study will be conducted at a private ophthalmological hospital in Brazil.

**Figure 1:**
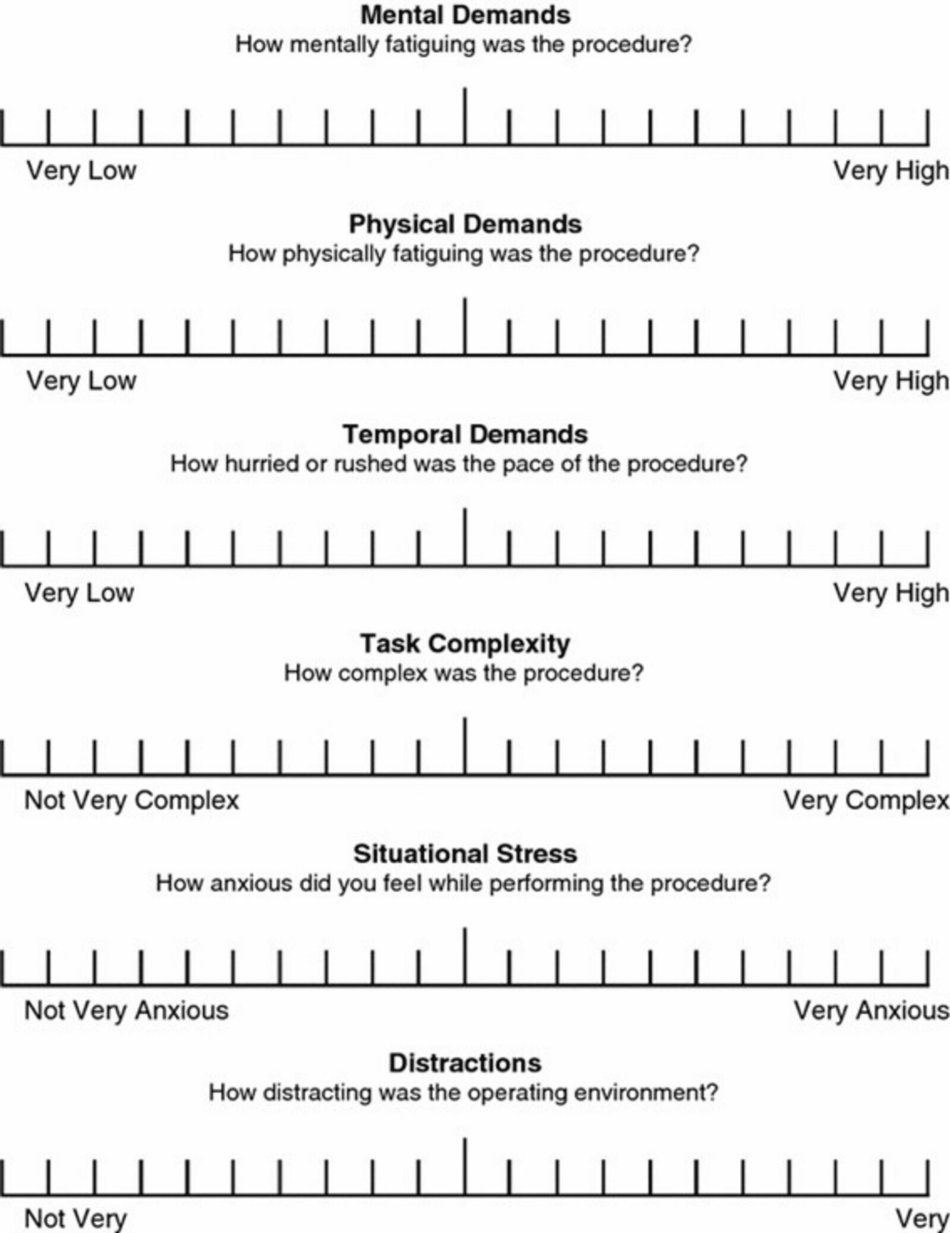
SURG-TLX Questionnaire

Patients will be monitored for sedation using the Ramsay Sedation Scale (Table 1), intraoperative difficulties will be assessed using the SURG-TLX Scale (Figure 1), and complications related to anesthesia and sedation will be recorded. Before the start of anesthesia and surgery, patients, surgeons, and anesthesiologists will be instructed on how to answer the questionnaires and what they measure to mitigate bias. The trial will ensure that both patients and the surgeon performing the surgeries are blinded to minimize bias. Randomization will be carried out in blocks of four using a 1:1 ratio. The trial is registered with the Brazilian Registry of Clinical Trials (ReBEC) under the identifier RBR-7c4c5jx.

**Table 1:** Ramsay’s Sedation Scale

Score	Definition
1	Patient awake, anxious, agitated or restless
2	Patient awake, cooperative, oriented and tranquil
3	Patient drowsy with response to commands
4	Patient asleep, brisk response to glabella tap or loud auditory stimuli
5	Patient asleep, sluggish to respond to stimuli
6	No response to firm nail-bed pressure or other noxious stimuli

The study has a defined non-inferiority margin of 10%. While the protocol includes robust measures to control for bias, a key limitation is the subjective nature of sedation assessments using scales and potential variability in patient responses to the drugs. Nonetheless, the design ensures that controlled comparisons between the sedative regimens can be reliably conducted.

Data to be collected**

Sex and systemic comorbidities: hypertension, diabetes mellitus, acute coronary syndrome, chronic kidney disease, asthma, heart failure, and psychiatric disorders.

Vital signs: oxygen saturation, heart rate, systolic blood pressure, and diastolic blood pressure will be measured before sedation, during surgery, and immediately before discharge.

Ramsay score: to be recorded before sedation, during surgery, and immediately after discharge (Table 1).

Additional sedation: the need for additional sedation during surgery will be noted.

Patient pain: pain during the procedure will be assessed using a Likert scale (Table 2).

**Table 2:** Post-Operative Pain Likert Scale

Score	Definition
1	Very Dissatisfied
2	Dissatisfied
3	Neutral
4	Satisfied
5	Very Satisfied

Patient preference: after the second surgery, the patient will choose which sedation they prefer: the first or second surgery.

SURG-TLX: surgery time, the number of cataract surgeries performed by the surgeon that day, and the specific number of the current surgery will be recorded (Figure 1).

Anesthesia-related complications: bradycardia, tachycardia, hypotension, hypertension, hypoxemia, apnea, myoclonus, oxygen saturation <90%, intraoperative agitation (Ramsay = 1 during surgery), postoperative nausea and vomiting, and others.

Sedation-related complications: aspiration, laryngospasm, intubation, hospitalization, mortality, and others.

Intraoperative complications: posterior capsule rupture (with or without vitreous loss), zonular injury, iris prolapse.

Participants

This study will involve patients undergoing phacoemulsification at HOBrasil, Salvador, Bahia, Brazil, between October 2024 and December 2024. We established Inclusion Criteria for Patients (Table 3), Exclusion Criteria for Patients (Table 4), Inclusion Criteria for Anesthetists (Table 5), and Inclusion Criteria for Surgeons (Table 6). 

**Table 3:** Inclusion Criteria for Patients

Patients undergoing phacoemulsification surgery.
Surgeries performed between October 2024 and December 2024 at HOBrasil, Salvador, Bahia, Brazil.

**Table 4:** Exclusion Criteria for Patients

Patients under 18 years of age.
Pregnant patients.
Patients with chronic pain syndrome.
Patients with hypersensitivity to any of the tested medications or topical anesthesia used.
Patients with communication difficulties (significant hearing loss, speech impairments, aphasias).
Patients with an ASA (American Society of Anesthesiologists) physical status classification of III-VI.

**Table 5:** Inclusion Criteria for Anesthetists

Anesthesiologist with a Specialist Title in Anesthesiology issued by the Brazilian Society of Anesthesiology.
At least ten years of experience in anesthesia for phacoemulsifications.
Participation in over 100 phacoemulsifications in the last year.

**Table 6:** Inclusion Criteria for Surgeons

Ophthalmologist with a Specialist Title in Ophthalmology issued by the Brazilian Council of Ophthalmology (CBO).
At least ten years of experience in performing phacoemulsifications.
Performed over 100 phacoemulsifications in the last year.

Withdrawal or drop-out criteria

Any patient can withdraw at any time for any reason in conditions that they wish to do so without any consequences, and the investigator will ensure that the patient’s care continues and that any reason for withdrawal is documented. 

Recruitment and screening

Recruitment will target patients undergoing phacoemulsification surgery at HOBrasil, Salvador, Bahia, Brazil, between October 2024 and December 2024. Eligible patients will be identified based on the study's inclusion and exclusion criteria. Screening will involve assessing the patient's age, medical history, and ASA physical status classification. Informed consent will be obtained after explaining the study procedures, risks, and potential benefits.

Randomization allocation and blinding

Following the application of eligibility criteria, patients eligible for phacoemulsification at HOBrasil, Salvador, Bahia, Brazil, will be randomly allocated to one of two groups. Group A will receive a combination of fentanyl with a benzodiazepine for sedation during the first eye surgery, followed by ketamine with a benzodiazepine for the second eye surgery. Group B will receive the reverse: ketamine with a benzodiazepine for the first eye and fentanyl with a benzodiazepine for the second. Randomization will be achieved using random number table generation software at a 1:1 ratio, employing a block randomization method with blocks of four to maintain balance between the groups. The number of patients in each block will remain undisclosed until the primary outcome analysis to preserve the secrecy of the randomization process. The randomization list will be integrated into the REDCap software, ensuring that the anesthetist is informed of the patient’s group allocation before each surgery while keeping the patient and the surgeon blinded to the sedative regimen. The sedative label will be concealed with tape to prevent unblinding.

Only the anesthetist, the randomization team, the data collection team, and the data analysis team will know which sedative is being administered. Both the surgeon and the patient will remain blinded to the type of sedation through the use of covered medication labels and strict confidentiality protocols. Unblinding will only occur if it is deemed necessary by the anesthetist, patient, or surgeon for patient safety. In such cases, only the sedative’s name will be revealed without disclosing the patient’s group. Any instance of unblinding will be reported, along with the reason for its occurrence, to ensure transparency and maintain the integrity of the trial.

Intervention

Before the surgery, patient eligibility will be reviewed to ensure that all inclusion criteria are met, including age, ASA classification, and consent. The procedure will be explained to the patient, and informed consent will be obtained. Monitoring equipment will be prepared, including an ECG, pulse oximeter, and non-invasive blood pressure monitor. The patient will be placed supine on the surgical bed, and oxygen will be administered through a nasal cannula at a rate of 4 L/min.

For sedation, Group Fentanyl will receive an intravenous dose of 0.71 µg/kg of fentanyl and 0.029 mg/kg of midazolam. Group Ketamine will receive an intravenous dose of 0.14 mg/kg of ketamine and 0.029 mg/kg of midazolam.

During the surgical procedure, sedation levels will be measured using the Ramsay Sedation Scale. Any intraoperative complications, vital signs instability, or need for additional sedation will be recorded.

After the surgery, the patient's vital signs will be monitored until discharge. Sedation quality will be assessed using a 1-5 Likert scale. In the event of intervention on the second eye, the patient will be asked which sedation they prefer, the one from the first or the second surgery. The patient will be discharged once an Aldrete score of ≥9 is achieved.

Study timeline

**Figure 2:**
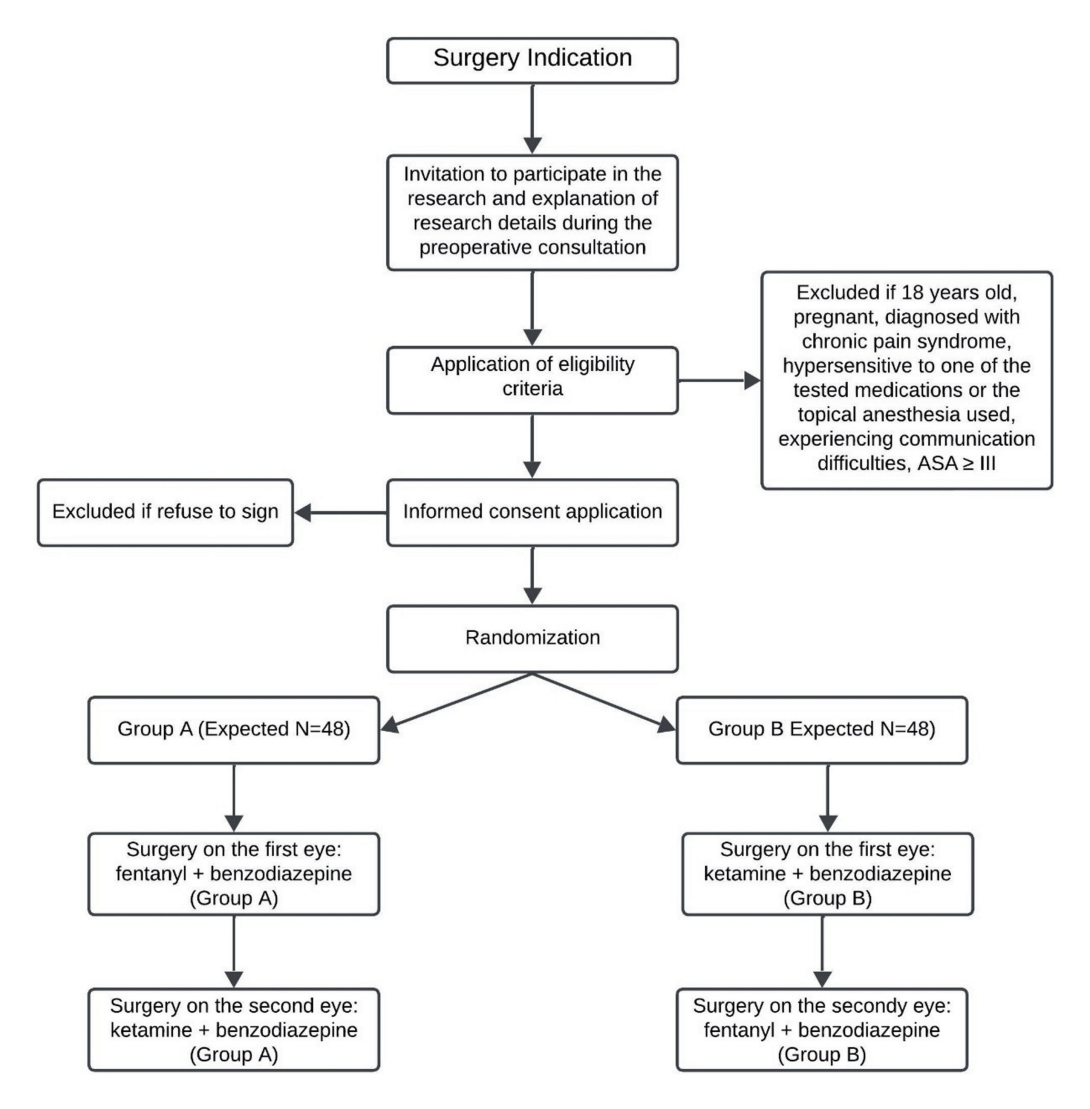
Study Chronology

Figure 2 shows the study chronology. 

Outcome assessment

Primary Outcome

The primary outcome is to demonstrate that sedation with ketamine combined with midazolam is non-inferior to fentanyl combined with midazolam, as measured by the Ramsay Sedation Scale (Table 1). Patients in both groups should achieve a sedation score of 2 or 3, indicating they are responsive to commands but drowsy, ensuring a safe and comfortable procedure.

Secondary Outcomes

Complications: The occurrence of anesthesia-related complications, such as bradycardia, hypoxemia, and postoperative nausea and vomiting, is expected to be minimal. The ketamine group may experience distinct psychomimetic effects (e.g., hallucinations or dissociative experiences) that may compromise blinding. If any patients present with psychomimetic effects, that will be explicitly reported in the results, and the randomization will continue as previously established. 

Patient and surgeon satisfaction

High levels of satisfaction are anticipated in both groups. Patient-reported pain scores (using a 1-5 Likert scale) should reflect minimal discomfort during surgery. Surgeon discomfort, assessed by the SURG-TLX, is also expected to be minimal in both groups.

Unsuccessful outcomes

If the protocol is unsuccessful, it could be due to insufficient sedation (Ramsay score = 1), resulting in patient discomfort or movement, excessive sedation (Ramsay score ≥ 4), or respiratory depression. In such cases, the procedure may need to be paused, rescue medications administered, or patient management adjusted.

Data management and monitoring

The data management process will be streamlined using REDCap (Research Electronic Data Capture) software. REDCap is a secure, user-friendly platform designed for efficient data entry, storage, and analysis. Its functionalities include automated export procedures, audit trails, and role-based access controls, ensuring data integrity and confidentiality throughout the research.

To enhance privacy, data will be entered in a non-identifiable manner, utilizing unique codes for each outcome. This approach provides an additional layer of confidentiality while preserving the robust features of the REDCap platform.

Sample size calculation

With an expected success rate of 87% in the control group (Midazolam) and 82% in the experimental group (Ketamine), maintaining a non-inferiority margin of 10% and a power of 80%, 48 eyes in each group will be required to achieve the primary objective of the study. The expected rate of success with the anesthetics was based on previous studies by Adinehmehr et al. and Yağan et al. [19,20]. The crossover double-blinded randomized study was based on the work of Cheung et al. [21].

Statistical analysis plan

Data collected in this study will be analyzed using R 4.1.0 software (R Core Team, 2021). The Shapiro-Wilk test will be used to assess the distribution of variable groups. Descriptive statistics will be reported as mean ± standard deviation for continuous variables and as the number of patients with the corresponding percentage (%) for nominal variables.

For data exhibiting a normal distribution, a Student’s t-test will be applied to compare two categorical variables. If the data are not normally distributed, the Mann-Whitney U test will be used instead. Categorical variables will be compared using Chi-square analysis. Statistical significance will be defined by a p-value < 0.05. In cases of protocol non-adherence, a per-protocol analysis will be performed. Multiple imputations will be used to address missing data.

Non-inferiority will be demonstrated if the difference in sedation effectiveness between the two groups falls within the pre-specified non-inferiority margin of 10%. Continuous variables will be summarized as mean ± standard deviation, while categorical variables will be presented as percentages.

## Results

The BEKEF study's results are expected to significantly enhance our understanding of sedation strategies during phacoemulsification. This trial aims to determine whether ketamine combined with a benzodiazepine provides non-inferior sedation compared to fentanyl with a benzodiazepine. Demonstrating this could be beneficial given ketamine's potentially lower risk of respiratory depression and more stable hemodynamic profile compared to fentanyl. The study hypothesizes that both drug regimens will achieve effective sedation, as measured by the Ramsay Sedation Scale while maintaining patient comfort and safety.

Additionally, the study expects to identify minimal anesthesia-related complications across both groups, with slight variations potentially observed in the incidence of postoperative nausea and vomiting, particularly in the ketamine group. Both patient and surgeon satisfaction are expected to be high, with comparable pain scores and low levels of intraoperative difficulty, as measured by the SURG-TLX scale.

By offering evidence of ketamine’s non-inferiority to fentanyl in this context, the study seeks to inform clinical protocols for sedation in cataract surgery, potentially supporting the use of ketamine as an alternative sedative option, particularly for patients with specific hemodynamic considerations.​

## Discussion

The BEKEF study aims to provide comparative insights into the sedation strategies during phacoemulsification, particularly between ketamine-benzodiazepine and fentanyl-benzodiazepine regimens. While ketamine has been recognized for its analgesic properties and minimal respiratory depression, it is less frequently employed in ophthalmic surgeries, where fentanyl remains the more conventional sedative [12,13]. This trial explores whether ketamine is a non-inferior alternative to fentanyl, with the potential for better hemodynamic stability and patient comfort, thereby providing an evidence-based option for sedation in cataract surgery.

Our study design draws on the methodology of previous trials investigating sedative efficacy in surgical settings, including crossover designs and double-blinding to reduce bias [17,18]. In addition to measuring patient sedation, we aim to assess broader outcomes, such as patient and surgeon satisfaction and complication rates. Such outcomes are particularly important in cataract surgery, where excellent results and patient satisfaction are expected.

The study’s strengths include its randomized crossover design, ensuring each patient is their control, thus minimizing interpatient variability. Additionally, using established scales such as the Ramsay Sedation Scale and the SURG-TLX for intraoperative assessments ensures objective and validated measurements of sedation and surgical difficulties, respectively. However, limitations exist. As with other sedation trials, subjective patient experiences and responses to medications could introduce variability despite the use of standardized scales. Moreover, the lack of long-term follow-up on patient outcomes, such as post-operative cognitive function or long-term pain management, limits the scope of our conclusions. Due to the crossover design, carryover effects could bias the results. Therefore, each eye surgery will be spaced fifteen days apart to mitigate this potential bias.

This study will provide much-needed evidence on the comparative efficacy of fentanyl and ketamine for sedation in cataract surgery. Should the findings demonstrate ketamine's non-inferiority or superiority, it could pave the way for broader adoption of this sedative in ophthalmic practices, potentially improving patient comfort and surgical outcomes.

There are some potential limitations to this study. First, the trial will be conducted at a single ophthalmological hospital in Brazil, and the patient population may not fully represent the global or more diverse populations, potentially limiting the generalizability of the results to other regions or ethnic groups. Second, despite the use of established scales such as the Ramsay Sedation Scale, the subjective nature of sedation assessments may lead to variability in results. Lastly, the study’s relatively short follow-up period limits the ability to assess long-term outcomes, such as postoperative pain management or prolonged recovery effects, which could provide further insights into the comparative benefits of fentanyl and ketamine.

## Conclusions

This paper outlines the protocol for the BEKEF study. The results will address a critical gap in the ophthalmic anesthesia literature by providing physicians and anesthetists with evidence on the comparative effectiveness of ketamine versus fentanyl, both combined with benzodiazepine, as sedative agents in phacoemulsification. The findings will offer valuable insights into optimizing sedation protocols for cataract surgery, ensuring patient comfort and safety while potentially broadening sedation options available for this procedure. 
